# The Frontal Assessment Battery (FAB) effectively discriminates between MCI and dementia within the clinical *spectrum* of neurochemically confirmed Alzheimer’s disease

**DOI:** 10.3389/fpsyg.2022.1054321

**Published:** 2022-11-30

**Authors:** Edoardo Nicolò Aiello, Federico Verde, Ilaria Milone, Eleonora Giacopuzzi Grigoli, Antonella Dubini, Laura Carelli, Roberta Ferrucci, Alberto Priori, Antonia Ratti, Erminio Torresani, Nicola Ticozzi, Vincenzo Silani, Barbara Poletti

**Affiliations:** ^1^Department of Neurology and Laboratory of Neuroscience, IRCCS Istituto Auxologico Italiano, Milan, Italy; ^2^PhD Program in Neuroscience, School of Medicine and Surgery, University of Milano-Bicocca, Monza, Italy; ^3^Department of Pathophysiology and Transplantation, Dino Ferrari Center, Università degli Studi di Milano, Milan, Italy; ^4^Neurology Residency Program, Università degli Studi di Milano, Milan, Italy; ^5^Laboratory of Clinical Chemistry, Department of Laboratory Medicine, IRCCS Istituto Auxologico Italiano, Milan, Italy; ^6^Aldo Ravelli Center for Neurotechnology and Experimental Brain Therapeutics, Department of Health Sciences, International Medical School, University of Milan, Milan, Italy; ^7^ASST Santi Paolo e Carlo, San Paolo University Hospital, Milan, Italy; ^8^IRCCS Ca’ Granda Foundation Maggiore Policlinico Hospital, Milan, Italy; ^9^Department of Medical Biotechnology and Translational Medicine, Università degli Studi di Milano, Milan, Italy

**Keywords:** executive functioning, Alzheimer’s disease, mild cognitive impairment, cerebrospinal fluid, frontal assessment battery

## Abstract

**Background:**

This study aimed at testing the ability of the frontal assessment battery (FAB) to differentiate between patients with mild cognitive impairment (MCI) and dementia due to Alzheimer’s disease (AD), as well as comparing its discriminative power to that of the Mini-Mental State Examination (MMSE).

**Methods:**

The present retrospective cohort included *N* = 107 Aβ-positive patients diagnosed with either MCI due to AD (*N* = 40) or probable AD dementia (ADD; *N* = 67). A two-step multiple logistic regression (MLR) was run to predict an MCI vs. ADD diagnosis based on FAB scores. Within the baseline step, demographics, disease duration, MMSE scores, and information on cognitive phenotypes were entered, with the FAB being added within the second step. Receiver-operating characteristics analyses were also run to derive intrinsic and post-test diagnostics.

**Results:**

Within the baseline MLR step, only lower MMSE scores predicted the occurrence of ADD; by adding the FAB, which likewise was able to discriminate between MCI and ADD (*p* = 0.016), a significant increase in model fit was detected (*p* = 0.007). The diagnostic efficiency of the FAB (AUC = 0.85) was comparable (*p* = 0.583) to that of the MMSE (AUC = 0.82), also yielding good intrinsic and post-test diagnostics, which were comparable to those of the MMSE.

**Discussion:**

The FAB is a diagnostically sound screener to discriminate between MCI and ADD, independently of patients’ overall cognitive profile. In doing so, the FAB is comparable to the MMSE, and the complementation of the latter with the former is advisable in order to increase the accuracy in differentiating between MCI and ADD within screening sessions.

## Background

Executive functioning (EF) deficits are a feature of both mild cognitive impairment (MCI) (Reinvang et al., 2012) and dementia due to Alzheimer’s disease (AD) (Guarino et al., 2019), which negatively affect performance in everyday activities (Marshall et al., 2011; Amanzio et al., 2018), and, as a marker of cognitive involution overall (Kirova et al., 2015), independently predict the conversion from MCI to dementia (Rozzini et al., 2007; Jung et al., 2020). Therefore, the availability of sound cognitive screening tests for the early detection of EF deficits is clinically crucial (Kirova et al., 2015).

In this regard, the Frontal Assessment Battery (FAB) (Dubois et al., 2000), a widespread and easy-to-perform screening test, has proved effective in detecting EF deficits in both MCI and dementia due to AD (ADD) (Hurtado-Pomares et al., 2018). Moreover, it has been validated against AD-specific neuroradiological biomarkers (Kume et al., 2011; Nagata et al., 2011; Oshima et al., 2012) and shown to be predictive of functional outcomes (Ikezaki et al., 2020). Nevertheless, currently available evidence on the capability of the FAB to discriminate between MCI and ADD is limited to one report (Yamao et al., 2011) that addressed a relatively small sample (*N* = 48) of selected, clinically diagnosed amnestic MCI and early-stage patients with ADD. Hence, information that is fully representative of the AD *spectrum* is still lacking, albeit being potentially relevant at a diagnostic and prognostic level (Rozzini et al., 2007; Marshall et al., 2011; Yamao et al., 2011; Reinvang et al., 2012; Kirova et al., 2015; Amanzio et al., 2018; Guarino et al., 2019; Jung et al., 2020); this is particularly true for AD as biologically defined according to the 2018 National Institute on Aging-Alzheimer’s Association (NIA-AA) amyloidosis/tauopathy/neurodegeneration (ATN) framework (Jack et al., 2018).

Given the above premises, this study aimed at testing the ability of the FAB to differentiate between neurochemically confirmed MCI and ADD, also comparing its discriminative power to that of a gold-standard screener for ADD – i.e., the Mini-Mental State Examination (MMSE) (Mitchell, 2017).

## Materials and methods

### Participants

The present retrospective cohort included *N* = 107 Aβ-positive patients diagnosed with either MCI due to AD (*N* = 40) (Albert et al., 2011) or probable ADD (*N* = 67), referred to the Department of Neurology, IRCCS Istituto Auxologico Italiano, Milan, Italy between June 2009 and April 2022, for whom FAB scores were available, and without evidence of (1) further neurodegenerative diseases or other neurological disorders impairing cognition, (2) ongoing organ/system failure, or (3) uncorrected sensory deficits.

All patients were administered a non-fixed, individual case-adapted cognitive battery (Supplementary Table 1) covering (1) attention, (2) executive functioning, (3) short-term memory, (4) long-term memory, (5) language, and (6) visuo-spatial/praxic abilities, which allowed us to classify them based on the type and number of impaired domains.

Patients with ADD—all of whom, at variance with MCI ones, had evidence of significant functional impairment in daily living—were further classified, based on *ad hoc* nosographic systems, as amnestic-predominant ADD (McKhann et al., 2011), logopenic variant-primary progressive aphasia (lvPPA) (Gorno-Tempini et al., 2011), posterior cortical atrophy (PCA) (Crutch et al., 2017), or behavioral variant-AD (bvAD) (Ossenkoppele et al., 2015).

### Materials

Appollonio et al.’s (2005) Italian version of the FAB was administered, and its scores were adjusted for age and education accordingly. MMSE scores (age- and education-adjusted) (Measso et al., 1993) were also available for all patients.

Patients’ ATN *status* was retrieved based on cerebrospinal fluid (CSF) neurochemical biomarkers according to the 2018 NIA-AA framework (Jack et al., 2018). Biomarker quantification and consequent classification were conducted as follows. For patients evaluated until September 2019, Aβ_42_, phosphorylated tau (P-tau_181_), and total tau (T-tau) were measured by using an enzyme-linked immunosorbent assay (ELISA). Patients were categorized as A+ or A− when having CSF Aβ_42_ levels ≤ or >647 pg/ml, respectively (Palmqvist et al., 2014), as T+ or T− when having CSF P-tau_181_ levels ≥ or <61 pg/ml (Vanderstichele et al., 2006), and as N+ or N− when having CSF T-tau ≥ or <three different age-determined cut-offs (≤50 years: 300 pg/ml; 51–70 years: 450 pg/ml; and >70 years: 500 pg/ml) (Sjögren et al., 2001). For patients evaluated from October 2019 onward, Lumipulse chemiluminescence enzyme immunoassays (CLEIAs) were used, with the Aβ_42_/Aβ_40_ – instead of the measurement of Aβ_42_ alone – defining A status (≤0.069, A+; >0.069, A−), and P-tau_181_ and T-tau having cut-offs of 56.5 and 404 pg/ml, respectively [the three cut-offs for CLEIAs are provided by the manufacturer and are comparable to those adopted in other studies (Gobom et al., 2022)].

### Statistics

A two-step multiple logistic regression (MLR) model was run to predict an MCI vs. ADD diagnosis based on FAB-adjusted scores (Appollonio et al., 2005). Within the baseline step, disease duration (months), ADD phenotype (typical – i.e., amnestic-predominant – vs. atypical – i.e., lvPPA, PCA, and bvAD), MMSE adjusted scores (Measso et al., 1993), amnestic vs. non-amnestic status, extra-mnestic, non-executive/-attentive cognitive status (i.e., at least one domain impaired vs. unimpaired among language, short-term memory, or visuo-spatial/praxic abilities), executive/attentive cognitive status (at least one domain among attention and/or executive functioning impaired vs. unimpaired), and the total number of impaired domains (*range* = 0–6) were entered. The FAB was then entered into the second step in order to test its incremental validity. Within both steps, model fit was assessed *via* Akaike’s Information Criterion (AIC) and MLR-based efficiency statistics computed – i.e., sensitivity (Se), specificity (Sp), and area under the curve (AUC). The difference in fit between the two steps was tested *via* a χ^2^-statistic. Collinearity was diagnosed in the presence of a variance inflation factor (VIF) >10 and a tolerance index (TI) <0.1 (Midi et al., 2010).

In addition, the AUC of the FAB was compared to that of the MMSE *via* receiver-operating characteristics (ROC) analyses by means of DeLong’s test for paired ROC curves (Robin et al., 2011). Intrinsic – i.e., Se and Sp – and post-test diagnostics – i.e., positive and negative predictive values (PPV; NPV) and likelihood ratios (LR+; LR−) – of the FAB were also computed at the optimal cut-off identified through Youden’s *J* statistic.

Analyses were run with jamovi 2.3.12^1^ and R 4.1.0^2^; the significance level was set α = 0.05.

## Results

Table 1 shows MCI and ADD patients’ background and clinical variables.

**TABLE 1:** Patients’ background and clinical variables.

	MCI	ADD	*p*
***N***	40	67	–
Age (years)	74.7 ± 5.8 (59–83)	73.4 ± 7 (53–84)	0.44^c^
Sex (male/female)	24/16	29/38	0.094^d^
Disease duration (months)	34.2 ± 29.6 (1–120)	33.7 ± 24.7 (3–96)	0.761^c^
MMSE (adjusted scores)^a^	24.5 ± 2.5 (19.3–30)	20.4 ± 3.7 (10.7–27.5)	<0.001^c^
**ADD phenotype (%)**			
Amnestic-predominant	–	83.6	–
lvPPA	–	7.5	–
PCA	–	7.5	–
bvAD	–	1.5	–
Number of impaired domains (/6)	2.5 ± 1.4 (1–6)	4.5 ± 1.2 (2–6)	<0.001
Amnestic status (%)	90	97	0.127^d^
Executive/attentive impairment (%)	52.5	94	<0.001^d^
Attention	27.5	65.7	<0.001^d^
Executive functioning	45	92.5	<0.001^d^
Extra-mnestic, non-executive/-attentive impairment (%)	65	95.5	<0.001^d^
Language	32.5	77.6	<0.001^d^
Short-term memory	17.5	52.2	<0.001^d^
Visuo-spatial/praxic abilities	37.5	68.7	0.002^d^
Frontal Assessment Battery (adjusted scores)^b^	15.2 ± 2.73 (8.5–18)	11.5 ± 2.9 (4.4–17.5)	<0.001^c^
Defective scores (%)	22.5	69.7	<0.001^d^
ATN status (%)			0.188^d^
A+T+N+	52.5	73.1	–
A+T−N+	2.5	1.5	–
A+T+N−	15	7.5	–
A+T−N−	30	17.9	–
Abnormal P-tau_181_ levels (%)	67.5	80.6	0.126^d^
Abnormal T-tau levels (%)	55	74.6	0.036^d^

ATN, amyloidosis/tauopathy/neurodegeneration framework (Sjögren et al., 2001); ADD, Alzheimer’s disease dementia; MCI, mild cognitive impairment; bvAD, behavioral variant of AD; lvPPA, logopenic variant of primary progressive aphasia; PCA, posterior cortical atrophy; P-tau_181_, tau phosphorylated at residue 181; T-tau, total tau. ^a^Measso et al. (1993); ^b^Appollonio et al. (2005); ^c^Mann–Whitney U-statistic; ^d^χ^2^-statistic.

Within the baseline MLR step (AIC = 85.9; AUC = 0.93; Se = 0.9; Sp = 0.75), only lower MMSE scores predicted the occurrence of ADD (*b* = −0.37; *z* = −3.04; *p* = 0.002). By adding the FAB within the second step (AIC = 80.5), a significant increase in model fit was detected [χ^2^(1) = 7.4; *p* = 0.007], with overall comparable, although slightly better, MLR-based diagnostics (AUC = 0.94; Se = 0.88; Sp = 0.8). Within such a step, lower FAB scores were predictive of the occurrence of ADD (*b* = −0.49; *z* = −2.42; *p* = 0.016) – with the MMSE being the only other variable yielding significance (*b* = −0.34; *z* = −2.55; *p* = 0.011). Within both the MLR steps, no collinearity was diagnosed (VIF ≤ 2.59; TI ≤ 0.95). The full results of both models are provided in Supplementary Tables 2, 3.

When tested within single-test ROC analyses, the diagnostic efficiency of the FAB (AUC = 0.85; SE = 0.04; CI 95% [0.74, 0.9]) was comparable (*z* = 0.55; *p* = 0.583) to that of the MMSE (AUC = 0.82; SE = 0.04; CI 95% [0.77, 0.92]). At the optimal cut-off of <12.95 (*J* = 0.55), the FAB yielded good intrinsic (Se = 0.67; Sp = 0.88) and post-test diagnostics (PPV = 0.9; NPV = 0.61; LR+ = 5.37; LR− = 0.38), which were overall comparable to those of the MMSE (*J* = 0.54; Se = 0.81; Sp = 0.73; PPV = 0.83; NPV = 0.69; LR + = 2.93; LR− = 0.27).

## Discussion

The present study demonstrates that the FAB is a diagnostically sound screener for discriminating between MCI and ADD in a cohort of Aβ-confirmed patients representative of the AD *spectrum*.

Indeed, the FAB showed good both intrinsic and post-test features in doing so, also having a diagnostic efficiency that was overall comparable to that of the MMSE – the latter being widely acknowledged as a gold-standard screener for ADD (Mitchell, 2017). Herewith, an age- and education-adjusted (Appollonio et al., 2005) cut-off of <12.95 is proposed for discriminating between MCI and ADD patients with CSF-confirmed evidence of underlying AD pathophysiology. Notably, such an adjusted score falls below the normality threshold of <13.4 proposed by Appollonio et al. (2005), this being in line with the fact that the cut-off proposed by the present study has to be addressed within the context of differentiating between two clinical conditions.

Furthermore, findings herewith show that the supplementation of the MMSE with the FAB results in a significantly higher power as to the discrimination between MCI and ADD – in agreement with a previous report, which, however, addressed the two screeners to the identification of ADD or its differentiation from non-AD dementias (Kim et al., 2014). Therefore, considering the relatively limited total amount of time that would be required (approximately 20 min), it might be reasonable to perform both batteries during the first evaluation of AD-*spectrum* patients.

Of note, the diagnostic efficiency of the FAB *per se* and its incremental validity against the MMSE alone apply to the whole *spectrum* of AD cognitive profiles – including amnestic features, which are characteristic of both MCI and ADD and predominant in the present cohort – and atypical ADD presentations (i.e., lvPPA, PCA, and bvAD). Taken together, such findings align with the notion of EF deficits being by themselves predictive of an overall greater disease severity/advanced disease across the AD *spectrum* (Rozzini et al., 2007; Kirova et al., 2015; Jung et al., 2020) – i.e., independently of other cognitive features, including long-term memory impairment (Rozzini et al., 2007).

Finally, a number of limitations of the present study have to be acknowledged. First, in order to control for the heterogeneity of cognitive/CSF data that had been originally collected for clinical purposes, a set of categorical classifications have been herewith adopted – this inherently implies a partial loss of information. Nevertheless, the availability of normative cut-offs for both cognitive and CSF measures, as well as the fact that diagnostic judgments were formulated by clinicians with experience in the field of cognitive disorders, likewise grant a sufficient degree of validity, and thus generalizability, of such classifications. On the other hand, it cannot be ruled out that an alternative screening test exploring another single cognitive domain – e.g., language – could perform equally well in discerning dementia from MCI due to AD: this issue was beyond the scope of the present investigation but deserves examination in the setting of a further, comparative study. Second, the present study does not include genetic, neuroimaging, and follow-up clinical information (e.g., related to MCI-dementia conversion): thus, future investigations are advisable that address also such data in relation to FAB scores within the AD *spectrum*.

## Conclusion

In conclusion, the FAB is a diagnostically sound screener to discriminate between MCI and ADD, independently of patients’ overall cognitive profile. In doing so, the FAB is comparable to the MMSE, and the complementation of the latter with the former is advisable in order to increase the accuracy in discriminating between MCI and ADD within screening sessions.

## Data availability statement

The raw data supporting the conclusions of this article are accessible, upon reasonable request of interested researchers to the corresponding author, at the following repository: https://zenodo.org/record/7288073#.Y2USunbMK3A.

## Ethics statement

The studies involving human participants were reviewed and approved by the Ethics Committee of IRCCS Istituto Auxologico Italiano (I.D: 2021_05_18_04). The patients/participants provided their written informed consent to participate in this study.

## Author contributions

EA: conceptualization, analyses, drafting, and revision. IM, EG, and LC: data collection and revision. AD, AR, NT, ET, and VS: resources and revision. RF and AP: revision. FV and BP: resources, conceptualization, drafting, and revision. All authors contributed to the article and approved the submitted version.

## Footnotes

1
https://www.jamovi.org/

2
https://www.r-project.org/
