# Supplementary material for: The Frontal Assessment Battery (FAB) effectively discriminates between MCI and dementia within the clinical spectrum of neurochemically confirmed Alzheimer’s disease
Source: Front Psychol. 2022 Nov 30;13:1054321. doi: 10.3389/fpsyg.2022.1054321 (PMC9748699; doi:10.3389/fpsyg.2022.1054321)
Supplement: Supplementary file 1 [file Table_1.DOCX]

| **Supplementary Table 1.** Cognitive battery for the cognitive phenotyping of patients. | |
| --- | --- |
| **Domain** | **Reference for normative data** |
| **Global cognitive efficiency** |  |
| Mini-Mental State Examination | Measso *et al.* (1993). *Developmental Neuropsychology, 9,* 77-85. |
| **Attention** |  |
| Digit Cancellation Task | Spinnler, H., & Tognoni, G. (1987). *Neurological Sciences, 6*, S1-S120. |
| Trail-Making Test-A | Giovagnoli, A. *et al.* (1996). *Neurological Sciences*, *17*, 305-309. |
| Trail-Making Test-B | Giovagnoli, A. *et al.* (1996). *Neurological Sciences*, *17*, 305-309. |
| Trail-Making Test-B-A | Giovagnoli, A. *et al.* (1996). *Neurological Sciences*, *17*, 305-309. |
| **Executive functioning** |  |
| Frontal Assessment Battery | Appollonio, I. *et al.* (2005). *Neurological Sciences*, *26*, 108-116. |
| Cognitive Estimation Task | Della Sala, S. *et al.* (2003). *Neurological Sciences*, *24*, 10-15. |
| Raven Colored Progressive Matrices | Basso, A. *et al.* (1996). *Functional Neurology*, *2*, 189-194. |
| Stroop Test (Short Form) | Caffarra, P. *et al.* (2002). *Nuova Rivista di Neurologia*, *12*, 111-115. |
| Phonemic Verbal Fluency | Novelli, G. *et al.* (1986). *Archivio di Psicologia, Neurologia e Psichiatria*, *4*, 477-506. |
| Tower of London | Shallice, T., McCarthy, R. (1982). *Philosophical Transaction of the Royal Society of London, 298*, 199–209 |
| **Language** |  |
| Token Test | Spinnler, H., & Tognoni, G. (1987). *Neurological Sciences*, *6*, S1-S120. |
| Boston Naming Test | Kaplan, E. *et al.* (1983). *Boston Naming Test*. Lea & Febiger. |
| Naming of living and non-living objects | Sartori, G. & Job, R. (1988). *Cognitive Neuropsychology*, *5*, 105-132. |
| Semantic Verbal Fluency | Novelli, G. *et al.* (1986). *Archivio di Psicologia, Neurologia e Psichiatria*, *4*, 477-506. |
| **Long-term memory** |  |
| Babcock Story Recall Test | Novelli, G. *et al.* (1986). *Archivio di Psicologia, Neurologia e Psichiatria*, *47*, 278-296. |
| Rey-Osterrieth Complex Figure - Delayed Recall | Caffarra, P. *et al.* (2002). *Neurological Sciences*, *22*, 443-447. |
| Free and Cued Selective Reminding Test | Frasson *et al.* (2011). *Neurological Sciences, 32*, 1057-1062. |
| Coupled Word List Recall | Novelli, G. *et al.* (1986). *Archivio di Psicologia, Neurologia e Psichiatria*, *47*, 278-296. |
| **Short-term memory** |  |
| Forward Digit Span | Monaco, M. *et al.* (2013). *Neurological Sciences*, *34*, 749-754. / Orsini *et al.* (1987) *Neurological Sciences*, *8*, 539-548. |
| Forward Corsi Span | Monaco, M. *et al.* (2013). *Neurological Sciences*, *34*, 749-754. / Orsini *et al.* (1987) *Neurological Sciences*, *8*, 539-548. |
| Backward Digit Span | Monaco, M. *et al.* (2013). *Neurological Sciences*, *34*, 749-754. |
| Backward Corsi Span | Monaco, M. *et al.* (2013). *Neurological Sciences*, *34*, 749-754. |
| **Visuo-spatial and praxic abilities** |  |
| Design Copy | Spinnler, H., & Tognoni, G. (1987).  *Neurological Sciences*, *6*, S1-S120. |
| Rey-Osterrieth Complex Figure – Copy | Caffarra, P. *et al.* (2002). *Neurological Sciences*, *22*, 443-447. |
| Street’s Completion Test | Spinnler, H., & Tognoni, G. (1987). *Neurological Sciences*, *6*, S1-S120. |
| Clock Drawing Test | Mondini *et al.* (2003). *Esame Neuropsicologico Breve 2.* Raffaello Cortina Editore. |

| **Supplementary Table 2.** Results of the first step of the MLR model (*i.e.*, without the FAB) | | | | | | | | | |
| --- | --- | --- | --- | --- | --- | --- | --- | --- | --- |
| **Predictor** | | ***b*** | | ***SE*** | | ***z*** | | ***p*** | |
| Intercept |  | 6.97418 |  | 3.4941 |  | 1.99598 |  | 0.04594 |  |
| Number of impaired domains |  | 0.61374 |  | 0.3755 |  | 1.63445 |  | 0.10216 |  |
| Amnestic *status*: |  |  |  |  |  |  |  |  |  |
| Non-amenstic *vs.* amnestic |  | -19.04779 |  | 2204.4866 |  | -0.00864 |  | 0.99311 |  |
| Executive/attentive *status*: |  |  |  |  |  |  |  |  |  |
| Normal *vs.* Impaired |  | -0.60097 |  | 0.9999 |  | -0.60101 |  | 0.54783 |  |
| Extra-mnestic, non-executive/attentive cognitive *status*: |  |  |  |  |  |  |  |  |  |
| Normal *vs.* Impaired |  | -0.57100 |  | 1.0683 |  | -0.53448 |  | 0.59301 |  |
| Disease duration (months) |  | -0.00528 |  | 0.0106 |  | -0.49748 |  | 0.61885 |  |
| Mini-Mental State Examination (adjusted scores) |  | -0.36775 |  | 0.1211 |  | -3.03646 |  | 0.00239 |  |
| Azlheimer’s disease dementia atypical variant: |  |  |  |  |  |  |  |  |  |
| Yes *vs.* No |  | 35.76403 |  | 3187.1223 |  | 0.01122 |  | 0.99105 |  |
| **Notes.** MLR=multiple logistic regression; FAB=Frontal Assessment Battery. *b* coefficients represent the odds ratio  of dementia over mild cognitive impairment. | | | | | | | | | |
|  | | | | | | | | | |

| **Supplementary Table 3.** Results of the second step of the MLR model (*i.e.*, with FAB) | | | | | | | | | |
| --- | --- | --- | --- | --- | --- | --- | --- | --- | --- |
| **Predictor** | | ***b*** | | ***SE*** | | ***z*** | | ***p*** | |
| Intercept |  | 14.068 |  | 4.8421 |  | 2.90530 |  | 0.00367 |  |
| Number of impaired domains |  | 0.310 |  | 0.3994 |  | 0.77729 |  | 0.43699 |  |
| Amnestic *status*: |  |  |  |  |  |  |  |  |  |
| Non-amenstic *vs.* amnestic |  | -20.737 |  | 2093.5434 |  | -0.00991 |  | 0.99210 |  |
| Executive/attentive *status*: |  |  |  |  |  |  |  |  |  |
| Normal *vs.* Impaired |  | 0.139 |  | 1.1210 |  | 0.12367 |  | 0.90158 |  |
| Extra-mnestic, non-executive/attentive cognitive *status*: |  |  |  |  |  |  |  |  |  |
| Normal *vs.* Impaired |  | -0.786 |  | 1.0992 |  | -0.71511 |  | 0.47454 |  |
| Disease duration (months) |  | 8.57e-4 |  | 0.0118 |  | 0.07257 |  | 0.94215 |  |
| Mini-Mental State Examination (adjusted scores) |  | -0.344 |  | 0.1351 |  | -2.54830 |  | 0.01082 |  |
| Azlheimer’s disease dementia atypical variant: |  |  |  |  |  |  |  |  |  |
| Yes *vs.* No |  | 37.524 |  | 3006.7122 |  | 0.01248 |  | 0.99004 |  |
| Frontal Assessment Battery (adjusted scores) |  | -0.494 |  | 0.2043 |  | -2.41711 |  | 0.01564 |  |
| **Note.** MLR=multiple logistic regression; FAB=Frontal Assessment Battery. *b* coefficients represent the odds ratio  of dementia over mild cognitive impairment. | | | | | | | | | |
